# A Nationwide Cross-Sectional Survey of Knowledge, Attitudes, and Practices on Rabies in Saudi Arabia

**DOI:** 10.3390/tropicalmed11020055

**Published:** 2026-02-17

**Authors:** Ebtisam Bakhsh, Rasha Doumi, Najd Alqahtani, Shahad Althubiti, Jana Hagr, Abeer Alnujide, Shouq Alobaid, Jana Allaboon, Shatha Alotaibi, Duaa Aljuhaymi, Maha Alotaibi, Abdullah Assiri

**Affiliations:** 1 Department of Internal Medicine, College of Medicine, Princess Nourah bint Abdulrahman University, Riyadh 11564, Saudi Arabia; 2 Department of Family and Community Medicine, College of Medicine, Princess Nourah bint Abdulrahman University, Riyadh 11564, Saudi Arabia; rbdoumi@pnu.edu.sa; 3 College of Medicine, Princess Nourah bint Abdulrahman University, Riyadh 11564, Saudi Arabia; najdfahad3@outlook.com (N.A.); shahadfaizz@gmail.com (S.A.); janawyhagr@hotmail.com (J.H.); abeeralnujide@gmail.com (A.A.); shouqkhalidalobaid@gmail.com (S.A.); janaallaboon5@gmail.com (J.A.); shatha.aye@gmail.com (S.A.); duaabdulaziz9@gmail.com (D.A.); maha2001otb@gmail.com (M.A.); 4 Ministry of Health, Riyadh 11176, Saudi Arabia; abdullahm.asiri@moh.gov.sa

**Keywords:** rabies, knowledge, attitudes, practices, public health, Saudi Arabia, zoonotic diseases

## Abstract

Rabies remains a fatal yet preventable zoonotic disease, and understanding population-level knowledge, attitudes, and practices (KAP) is essential to guide national elimination strategies. This nationwide cross-sectional study assessed rabies-related KAP among 2116 residents across all major regions of Saudi Arabia. An online validated questionnaire measured sociodemographic characteristics and KAP indicators. Descriptive and inferential statistics, including logistic regression, were used to identify predictors of good KAP outcomes. Overall, more than half of participants demonstrated poor knowledge (54.9%), particularly regarding rabies etiology, transmission, and essential post-exposure wound care, while attitudes toward prevention were overwhelmingly positive (92%). Despite this, several unsafe practices persisted, including interaction with stray animals and use of traditional remedies. Good knowledge was significantly associated with higher income, pet ownership, and residency in the Central region, whereas younger age and Northern residency predicted poorer practices. Mediation analysis suggested that knowledge may partly explain (mediate) the association between sociodemographic characteristics and reported preventive practices; however, causal inference is limited by the cross-sectional design. These findings demonstrate substantial knowledge and behavioral gaps despite favorable attitudes and highlight the need for culturally tailored educational interventions, improved access to post-exposure prophylaxis, and strengthened One Health strategies to support rabies elimination in Saudi Arabia.

## 1. Introduction

Rabies remains one of the oldest yet persistently neglected zoonotic diseases, with profound implications for public health, veterinary systems, and socio-economic development worldwide. Despite being entirely preventable, rabies continues to cause an estimated 59,000 human deaths annually, predominantly in low- and middle-income countries in Asia and Africa, where health infrastructure, animal vaccination programs, and community awareness are insufficiently robust [1,2,3]. Transmission occurs mainly through the bite or scratch of infected animals, particularly domestic dogs, which are responsible for more than 95% of human rabies cases globally [4,5]. Once clinical symptoms emerge, the disease is almost invariably fatal, underscoring the critical importance of prevention through timely post-exposure prophylaxis (PEP) and mass dog vaccination [6].

From a global health policy perspective, rabies is emblematic of the “One Health” approach, which emphasizes the interconnection between human, animal, and environmental health. The World Health Organization (WHO), the Food and Agriculture Organization (FAO), and the World Organisation for Animal Health (WOAH) launched the “Zero by 30” initiative in 2018, setting an ambitious target of eliminating human deaths from dog-mediated rabies by 2030 [7]. This strategy calls for strengthening national health systems, scaling up canine vaccination to achieve at least 70% coverage, ensuring universal access to affordable PEP, and enhancing community education about rabies prevention [8,9]. Yet, the pathway toward achieving this target remains challenging due to persistent gaps in surveillance, intersectoral collaboration, and public knowledge and practices [10].

Operationalizing One Health for rabies requires formal coordination across human health (bite management/PEP), veterinary services (animal vaccination and outbreak investigation), municipalities (stray animal management), and environmental/wildlife stakeholders (wild carnivore surveillance) [11]. In Saudi Arabia, where One Health coordination is often highlighted for priority threats such as AMR and emerging infections, rabies elimination would benefit from similarly structured cross-sector governance, shared data systems, and joint risk communication [12].

In the Arabian Peninsula, rabies epidemiology also includes wildlife transmission cycles. Evidence from regional reviews and Saudi animal surveillance indicates that foxes and other wild carnivores contribute to maintenance and spillover risk, which has implications for surveillance, veterinary vaccination strategies, and public risk communication beyond stray dog/cat exposure alone [13,14]. Sporadic outbreaks in both animals and humans have been documented in several Gulf countries, underscoring the need for integrated approaches that address cultural practices, animal control policies, and public awareness [15]. Saudi Arabia, as the largest country in the Arabian Peninsula, presents a particularly important case study given its diverse geography, large expatriate workforce, and unique mix of urban and rural populations.

National surveillance indicates that animal bites continue to be reported across the Kingdom, underscoring the importance of ensuring timely access to post-exposure prophylaxis (PEP), including rabies vaccine and rabies immunoglobulin, through Ministry of Health services. In Saudi Arabia, PEP is administered within MoH care pathways, primarily via public hospitals and primary healthcare centers. However, access in practice depends on healthcare-seeking behavior after exposure, perceived risk, and the geographic availability of PEP services, which can be challenging in underserved or remote communities [16,17]. Rabies is a reportable (notifiable) condition in Saudi Arabia, and health facilities are required to notify suspected or confirmed cases, reinforcing the need for high-quality surveillance data and consistent clinical pathways for bite management and PEP delivery. However, confirmed human rabies cases remain rare, raising questions about under-diagnosis, reporting gaps, and public misconceptions about disease transmission. The coexistence of rising stray animal populations, inconsistent pet vaccination, and limited public knowledge creates a potential “silent reservoir” for rabies transmission that could undermine elimination efforts. In major urban centers such as Riyadh, Jeddah, and Dammam, reports of dog bites and public fear of stray animals have increased, highlighting the social salience of rabies as both a health and community safety issue [13].

Knowledge, attitudes, and practices (KAP) surveys have become an essential methodological tool to assess public readiness for rabies prevention programs. Studies from Africa, South Asia, and Latin America consistently reveal a paradox: while awareness that rabies is dangerous is widespread, detailed knowledge about transmission routes, incubation periods, and the importance of immediate wound washing and vaccination remains poor [18,19]. Misconceptions—such as reliance on herbal remedies or belief that rabies can be treated with antibiotics—are pervasive and often lead to fatal delays in seeking PEP [20]. Attitudinal factors also play a decisive role. Positive community attitudes toward dog vaccination, reporting of suspected rabid animals, and prompt healthcare-seeking can reinforce national elimination strategies, whereas indifference or fatalistic beliefs perpetuate risks [21]. Practices, however, frequently lag behind both knowledge and attitudes, as risky behaviors such as playing with stray animals or ignoring minor bite wounds remain common [22].

In Saudi Arabia, very limited research has explored the population’s KAP toward rabies. A few small-scale studies indicate low levels of rabies knowledge and suboptimal practices even among educated groups [23]. For instance, a survey in Riyadh reported that less than half of respondents knew that rabies is uniformly fatal without treatment, and significant proportions were unaware of the importance of vaccination after dog or cat bites [24]. Given the high proportion of youth in Saudi society, many of whom interact with pets and stray animals, understanding generational and regional differences in KAP is critical for designing targeted interventions. Moreover, the cultural diversity of the Kingdom, shaped by the presence of large numbers of expatriates, necessitates consideration of heterogeneous beliefs and behaviors regarding animal contact and healthcare-seeking [25].

The urgency of addressing rabies prevention in Saudi Arabia is reinforced by the broader goals of Vision 2030, which emphasizes strengthening preventive health, expanding veterinary services, and improving zoonotic disease surveillance. Integrating rabies elimination into these reforms could yield substantial health and economic benefits, particularly since cost-effectiveness studies from Tanzania and Indonesia have shown that mass dog vaccination is more sustainable and less expensive in the long run than reliance on PEP alone [26,27]. At the same time, public education campaigns tailored to local cultural and linguistic contexts are indispensable. For example, incorporating rabies awareness into school curricula, mosque-based health promotion, and social media platforms could reach younger populations more effectively than traditional approaches [28].

Recent policy attention to stray animal management in parts of the Kingdom may influence exposure patterns over time. Nevertheless, effective rabies prevention requires that any stray animal management be integrated with vaccination, surveillance, and humane animal welfare frameworks to avoid unintended public health and ethical harms [13,29].

### 1.1. Aim of the Study

The study aimed to explore the knowledge, attitudes, and practices (KAP) related to rabies among the Saudi population and to identify sociodemographic factors associated with these outcomes. By providing a large, geographically distributed online sample, the study highlights gaps that may hinder rabies prevention efforts, the study sought to highlight critical gaps that may hinder the achievement of rabies prevention and elimination goals within the framework of Saudi Arabia’s Vision 2030 and the global *Zero by 30* initiative.

### 1.2. Research Questions

1. What is the current level of knowledge, attitudes, and practices regarding rabies among the Saudi population?
2. Which sociodemographic factors (e.g., age, gender, education, income, region, and pet ownership) are associated with differences in knowledge, attitudes, and practices toward rabies prevention?

## 2. Materials and Methods

### 2.1. Study Design

This research employed a nationwide, cross-sectional survey design, a commonly used approach for Knowledge–Attitudes–Practices (KAP) assessment in public health research and for informing risk communication and intervention planning [30,31]. The design allowed for the collection of quantitative data within a relatively short period of time, providing a snapshot of rabies-related awareness and behaviors among diverse demographic groups across Saudi Arabia. The cross-sectional nature ensured the inclusion of participants from varied age groups, educational backgrounds, socioeconomic levels, and geographical regions, enabling the identification of patterns and associations that are critical for tailoring national health interventions.

### 2.2. Study Setting

The study was conducted across all five major geographical regions of Saudi Arabia: the Central, Eastern, Western, Northern, and Southern regions. Each region has distinct demographic, cultural, and socio-economic characteristics, offering an opportunity to capture regional disparities in rabies knowledge and practices. Urban centers such as Riyadh, Jeddah, and Dammam provided perspectives from densely populated metropolitan environments, while smaller towns and rural areas reflected experiences where contact with stray or domestic animals may be more common and healthcare access more limited. This wide coverage strengthened the geographic breadth of the sample; nonetheless, representativeness is limited by online convenience recruitment.

### 2.3. Sample and Sampling

The target population comprised adult residents of Saudi Arabia (≥18 years) who were able to read Arabic. This includes Saudi citizens and Arabic-speaking expatriate residents, reflecting the multilingual, multi-national population structure of the Kingdom. The survey link was distributed via popular social media platforms, including WhatsApp, Twitter (X), and Instagram, and a snowballing technique was employed by encouraging participants to share the link within their networks. This approach allowed rapid and broad recruitment, particularly among younger and urban populations, while also ensuring inclusion of participants from remote areas who had internet access. Sample size calculations using EpiTools, (Version: 0.5-10.1) with an assumed 50% response distribution, 95% confidence level, and 2% margin of error, indicated a minimum requirement of 865 participants. Ultimately, 2116 individuals completed the survey, far exceeding the required sample size and thereby enhancing statistical power for subgroup comparisons; however, as an online convenience sample, findings should be interpreted as nationwide in geographic coverage rather than nationally representative.

### 2.4. Data Collection Tools

Data were collected using a structured, self-administered questionnaire that was adapted from previously validated KAP surveys on rabies conducted internationally. The primary reference tools included the rabies KAP questionnaire developed by Ahmed et al. in Pakistan (BMC Public Health, 2020) and Tiwari et al. in India (PLoS Negl Trop Dis, 2019), both of which were designed to measure public awareness and behaviors regarding rabies prevention and control. The adaptation process ensured cultural appropriateness and contextual relevance for the Saudi population.

The questionnaire was organized into four main sections: (1) sociodemographic data (age, gender, education, marital status, occupation, income, region, and pet ownership); (2) knowledge of rabies, including etiology, transmission routes, clinical features, prevention, and outcomes; (3) attitudes toward rabies control, vaccination, and preventive behaviors; and (4) practices following potential exposure to suspected rabid animals. The knowledge section contained multiple-choice and true/false items, with each correct response awarded one point. A total knowledge score was calculated, and participants achieving the 60% threshold was selected based on commonly used Bloom’s cut-off approaches in KAP research, where scores ≥ 60% are often treated as at least sufficient/moderate-to-good knowledge, and <60% as poor. We adopted a dichotomized classification to support logistic regression modeling and interpretability”. The attitude section used Likert-scale items (agree, neutral, disagree) to evaluate respondents’ perspectives, with positive attitudes defined by overall favorable responses. The practices section assessed behaviors such as wound care, vaccination after bites, and interactions with stray animals, with responses categorized as good or risky practices.

The reliability and validity of the tool were carefully evaluated. Internal consistency was assessed using Cronbach’s alpha, which yielded satisfactory coefficients across domains (>0.75), indicating good reliability. Face and content validity were established through expert review by a panel of public health and infectious disease specialists in Saudi Arabia, who confirmed the relevance and comprehensiveness of the items. The questionnaire was first prepared in English and then translated into Arabic using a forward–backward translation method to ensure semantic equivalence. The initial translation and back-translation, followed by reconciliation was conducted by the research team. A pilot test involving 30 participants confirmed the clarity, readability, and cultural appropriateness of the Arabic version. Minor modifications were made based on feedback before full-scale deployment.

### 2.5. Data Collection Procedure

The survey was conducted online between December 2023 and January 2024. The electronic questionnaire was designed using Google Forms and optimized for both desktop and mobile devices to maximize accessibility. A brief introduction at the beginning of the survey explained the study objectives, confidentiality measures, and voluntary nature of participation. Participants were required to provide electronic informed consent before proceeding to the questionnaire. The survey link was shared through the investigators’ professional networks and public social media groups, with reminders issued weekly to enhance participation. Data were automatically captured in the secure Google Forms database and subsequently exported to SPSS (version 26) for analysis. To minimize duplicate entries, the survey settings were configured to limit participation to one response per account. No incentives were offered. Records were screened for obvious duplication patterns (identical demographics and response strings submitted within short intervals)

The survey link was shared through the investigators’ professional networks and public social media groups (WhatsApp, X/Twitter, and Instagram). Weekly reminders were posted during the data collection window (December 2023–January 2024) to the same channels to enhance reach and participation

### 2.6. Data Analysis

All statistical analyses were performed using IBM SPSS Statistics version 29. Descriptive statistics, including frequencies, percentages, means, and standard deviations, were used to summarize demographic variables and KAP outcomes. Inferential analyses were conducted to examine associations between sociodemographic variables and KAP scores. Chi-square tests were employed for categorical variables, while independent t-tests and one-way ANOVA were used to compare mean scores across groups where appropriate. Logistic regression models were developed to identify predictors of good knowledge, positive attitudes, and good practices, adjusting for potential confounders. A *p*-value < 0.05 was considered statistically significant.

As a non-probability online survey, nonresponse bias analyses such as early–late respondent comparison were not formally performed, which limits inference regarding differences between early and late participants. Future national studies should incorporate probability sampling frames and targeted recruitment of rural/low-connectivity communities to enable such assessments.

To reduce the likelihood of repeat participation, the Google Forms setting was configured to restrict submissions to a single response per authenticated account. In addition, we conducted post-collection screening for potential duplicates by examining submissions for highly similar response patterns (e.g., identical sociodemographic profiles combined with near-identical item-response strings submitted within a short time window). No incentives were offered, further reducing motivation for repeat entries. Nevertheless, as with most anonymous online surveys, we acknowledge that duplicate submissions cannot be eliminated with absolute certainty; this limitation is now stated explicitly.

### 2.7. Ethical Considerations

Ethical approval for the study was obtained from the Institutional Review Board (IRB) of Princess Nourah bint Abdulrahman University, Riyadh, Saudi Arabia (Approval No: IRB Log Number: 24-0111). All participants were informed about the purpose, procedures, and voluntary nature of the study. They were assured that no personal identifiers would be collected, and confidentiality of responses was guaranteed. Electronic informed consent was obtained before survey initiation, and participants were allowed to withdraw at any point without penalty. Data were stored securely with access restricted to the research team. The study adhered to the principles of the Declaration of Helsinki and complied with national ethical standards for human subject research.

## 3. Results

As shown in Table 1, the study sample (*n* = 2116) was predominantly young, female, and highly educated. Almost two-thirds of participants were younger than 25 years (63.6%), and only 6.1% were older than 45 years. Women constituted more than three-quarters of the sample (76.6%). Most respondents held a university degree (77.0%), while only 15.5% reported secondary school education or less, and 7.5% had postgraduate qualifications. Over half were students (57.8%), one quarter were employed (25.0%), and 17.2% were unemployed. Nearly three-quarters were unmarried (74.1%). Geographically, about one-third resided in the Central region (32.1%), followed by the Western (21.7%) and Eastern (17.1%) regions, with smaller proportions from the Northern (16.0%) and Southern (13.0%) regions. With respect to socioeconomic and exposure-related characteristics, most participants reported that their family income was “enough” (68.6%), with similar proportions indicating “enough and safe” (15.5%) or “not enough” (15.9%). The majority did not own pets (78.8%), although about half reported that their residential area contained stray animals (50.7%). Only 10.5% had ever been bitten by an animal, but more than one-third (37.8%) reported having heard of or known someone who had rabies, while 59.3% had no such experience and 12.9% were unsure

The demographic skew toward younger and female respondents likely reflects the online recruitment strategy and the higher engagement of students and young adults with social media-based survey dissemination. This pattern is common in web-based public health surveys and should be interpreted as a limitation rather than evidence of population structure. Importantly, the findings remain valuable because they identify knowledge and practice gaps in a highly connected group that can rapidly amplify information (or misinformation) through social networks.

Table 2 illustrates substantial gaps and inconsistencies in rabies-related knowledge among participants. While most respondents recognized that rabies is preventable (63.8%), fewer understood key details about its cause, transmission, and symptoms. Less than half correctly identified rabies as viral (41.7%), and nearly one-third were unsure about its infectious nature. Although dog bites were widely acknowledged as a primary transmission route (80.5%), awareness of other transmitting animals and symptoms in humans and animals was limited. Knowledge of the incubation period was especially poor, with two-thirds reporting “I don’t know”.

Post-exposure practices also reflected misconceptions: although 61.9% would seek hospital care, only 14.3% identified proper wound washing, and 20.8% were unsure what to do. Overall, more than half of participants (54.9%) demonstrated poor knowledge, highlighting critical gaps that may hinder timely recognition and appropriate response to rabies exposure

Table 3, shows that Attitudes were generally positive, with approximately nine in ten respondents supporting vaccination campaigns, stray animal control, and prompt medical consultation after bites. However, a 35.5% minimized the need for rabies vaccination after “mild” wounds, reflecting a dangerous misconception that could undermine prevention strategies.

Table 4 shows that, overall, participants reported generally favorable rabies-related practices but with important inconsistencies. Most respondents said they would *always* or *often* advise a bitten person to be vaccinated (90.2%) and would visit the hospital themselves after a bite (91.4%), suggesting good awareness of the need for post-exposure care. Similarly, three-quarters reported consistently keeping away from aggressive animals, and about three-quarters indicated that they wash bite wounds with soap and water for at least 15 min, which aligns with recommended first-aid practice.

However, several risky behaviors remain common. Nearly half of respondents stated that they *always* or *often* need someone to remind them to get vaccinated after a bite, implying limited self-initiated action. A sizeable minority reported playing with stray pets at least sometimes (45.4%), which increases exposure risk. Traditional practices also persist: more than one-third *always* or *often* use local herbs on the bite, and almost one quarter do nothing or delay action by allowing the wound to heal on its own.

Inferential testing revealed that several sociodemographic characteristics were significantly associated with participants’ KAP outcomes (Table 5). Higher income, pet ownership, and residency in the Central or Eastern regions were strongly associated with better rabies knowledge (*p* < 0.01). Younger participants (<25 years) and those from the Northern region had significantly poorer practices compared with their older and centrally located counterparts (*p* < 0.05). Multivariable logistic regression confirmed that income sufficiency (OR = 2.14, 95% CI: 1.62–2.84), pet ownership (OR = 1.73, 95% CI: 1.29–2.31), and residence in the Central region (OR = 1.56, 95% CI: 1.18–2.06) were independent predictors of good knowledge. Similarly, younger age (OR = 0.61, 95% CI: 0.46–0.82) and Northern residency (OR = 0.58, 95% CI: 0.43–0.77) were independent predictors of poor practices. Positive attitudes, however, were consistently high across groups, with only minor variation by age and income.

**Figure 1:**
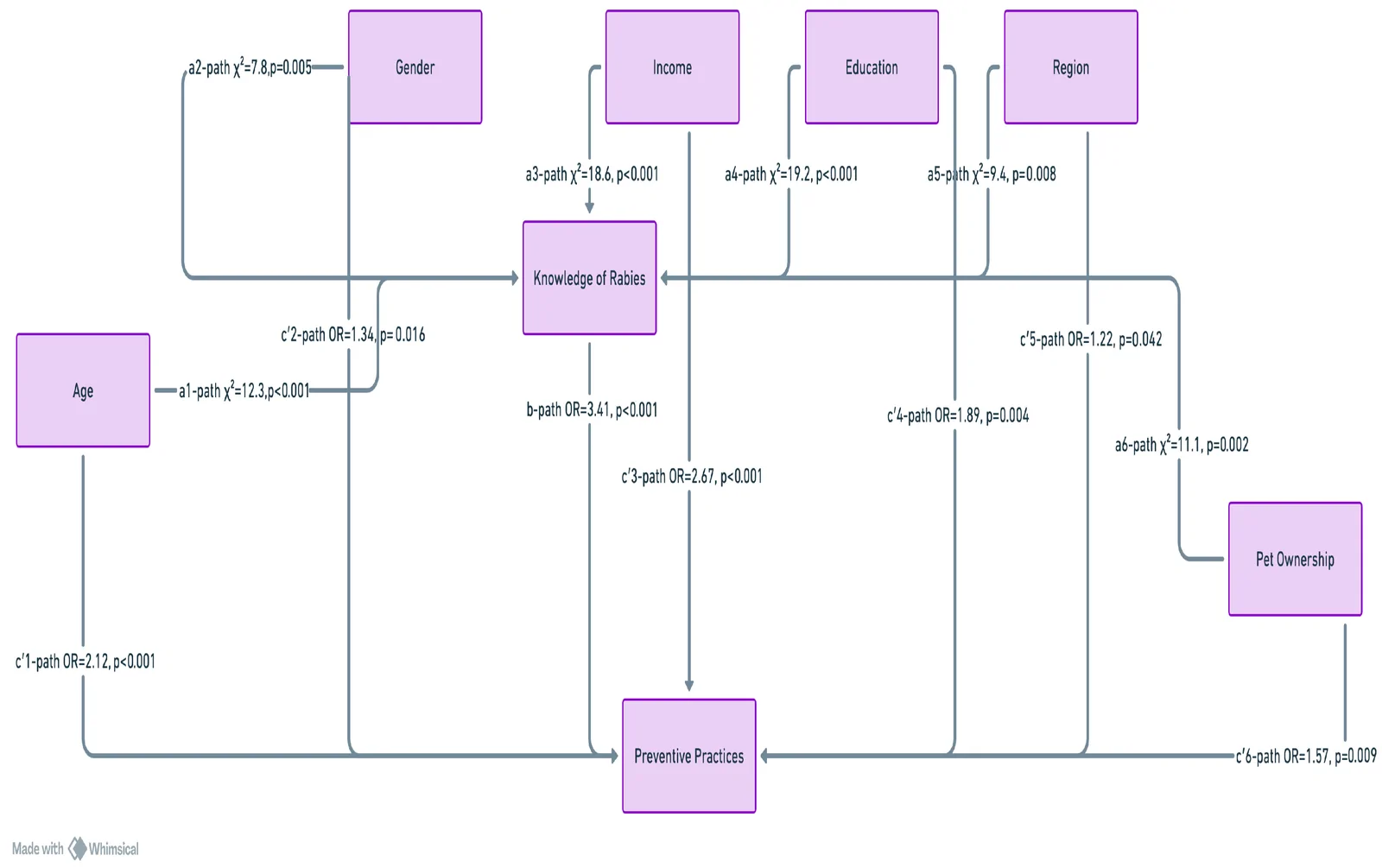
The mediation model of rabies knowledge.

The mediation model demonstrated that rabies knowledge significantly explained the pathway between sociodemographic factors and preventive practices (Figure 1). For example, participants with higher income were more than twice as likely to demonstrate good knowledge (OR = 2.14, 95% CI: 1.62–2.84), and those who owned pets showed a 73% greater likelihood of good knowledge (OR = 1.73, 95% CI: 1.29–2.31). Knowledge in turn strongly predicted safer practices, with participants who had good knowledge being over three times more likely to engage in preventive behaviors such as wound washing and timely hospital visits (OR = 3.41, *p* < 0.001). Conversely, younger individuals (<25 years) were 39% less likely to demonstrate good practices (OR = 0.61, 95% CI: 0.46–0.82), and residents of the Northern region were 42% less likely (OR = 0.58, 95% CI: 0.43–0.77). These findings confirm that while demographic characteristics exert direct effects on practices, much of their influence is mediated through knowledge. Interventions that close knowledge gaps—particularly targeting youth and populations in underserved regions—are therefore likely to yield the greatest improvements in preventive behaviors.

## 4. Discussion

This nationwide cross-sectional survey provides a detailed examination of knowledge, attitudes, and practices (KAP) related to rabies among the Saudi population. The findings highlight a paradox well documented in health psychology and behavioral medicine: although attitudes toward rabies prevention were overwhelmingly positive, knowledge gaps and risky practices persisted. This discordance between attitudes and behaviors underscores the need to address not only awareness but also the psychosocial determinants of health behavior change.

### 4.1. Knowledge Gaps and Misconceptions

Despite widespread awareness of its existence. Specifically, misconceptions about rabies transmission and prevention were common; only 36.9% correctly identified vaccination as the main preventive measure,. Similar misconceptions have been observed in South Asia and Africa, where large-scale surveys revealed that awareness of rabies as a fatal disease did not necessarily translate into accurate understanding of transmission routes or preventive strategies [32]. This reflects what health psychologists describe as a superficial awareness, in which general recognition of a health threat does not translate into effective protective action [33].

From a cognitive–behavioral perspective, incomplete knowledge limits the ability to assess risk accurately, often leading to maladaptive coping strategies such as reliance on traditional remedies. Indeed, 36.2% of respondents in this study reported using herbal local treatments after animal bites. Reliance on such culturally embedded practices has also been documented in Mozambique and Bhutan, where delays in seeking medical treatment frequently resulted in preventable deaths [34]. The persistence of such behaviors illustrates how cultural schemas and health beliefs can override biomedical recommendations, even when individuals express supportive attitudes toward vaccination and hospital care [35].

### 4.2. Attitudes Versus Practices

The study revealed that 92.1% of respondents endorsed positive attitudes toward rabies prevention, such as supporting pet vaccination and recognizing the importance of hospital visits after bites. Yet, nearly one-third reported playing with stray animals and more than one-third applied traditional remedies to wounds. This mismatch between attitudes and practices reflects the long-recognized “intention–behavior gap” in health psychology, whereby favorable attitudes do not always predict preventive behaviors [36]. According to the Theory of Planned Behavior, attitudes interact with subjective norms and perceived behavioral control to shape action [37]. In the Saudi context, where strong familial and community traditions influence health-seeking, perceived barriers such as stigma, accessibility, or mistrust of services may weaken the translation of intentions into behavior.

Furthermore, the Health Belief Model suggests that individuals weigh perceived susceptibility and perceived severity against perceived benefits and barriers before taking action [38]. While participants recognized rabies as severe, the relatively low perceived susceptibility—given the rarity of confirmed human cases in Saudi Arabia—may have contributed to complacency in adopting safe practices. Such findings highlight the need for interventions that increase risk salience and emphasize susceptibility, particularly among younger individuals who demonstrated the poorest preventive practices [39].

### 4.3. Sociodemographic Influences

Demographic variables significantly shaped KAP outcomes. Higher income and pet ownership were associated with better knowledge, while younger age and residence in the Northern region predicted poorer practices. These patterns align with international evidence showing that socioeconomic status and direct exposure to animals are critical determinants of rabies literacy [40]. For instance, studies from India and Tanzania revealed that individuals with higher education and income were more likely to seek timely post-exposure prophylaxis [41].

The vulnerability of younger populations in this study is particularly concerning, given that more than 60% of the sample was under 25 years old. Younger respondents were significantly less likely to report safe practices such as wound washing or hospital visits. This finding is consistent with behavioral science literature indicating that adolescents and young adults are more prone to risk-taking behaviors, often underestimating the seriousness of health threats [42]. Targeted interventions for this age group—such as integrating rabies education into school curricula or leveraging social media campaigns—may therefore be particularly effective.

Regional disparities also emerged, with participants in the Northern region demonstrating both lower knowledge and poorer practices compared to those in the Central and Eastern regions. Similar geographical inequalities in rabies awareness have been documented in Madagascar and sub-Saharan Africa, where rural areas had less access to health information and post-exposure prophylaxis (PEP) [43]. In Saudi Arabia, such disparities may reflect differences in healthcare infrastructure, veterinary services, and regional investment in public health campaigns.

### 4.4. Behavioral and Policy Implications

The findings have important implications for designing interventions. First, knowledge emerged as a significant mediator of the relationship between sociodemographic characteristics and practices. For example, participants with higher income or pet ownership were more likely to have good knowledge, which in turn predicted safer practices. This mediation effect underscores the central role of knowledge enhancement in bridging the gap between demographics and behavior. Interventions that strengthen rabies literacy—through culturally adapted, community-based programs—could therefore yield substantial improvements in preventive practices.

Second, psychosocial and behavioral insights must guide intervention strategies. Simply increasing awareness is insufficient; interventions should also address perceived barriers, cultural norms, and self-efficacy. For instance, studies in Ethiopia and Nigeria demonstrated that community education programs combining biomedical information with culturally sensitive messages were more effective than awareness campaigns alone. In Saudi Arabia, mosque-based health promotion, school engagement, and collaboration with social media influencers could enhance both knowledge and behavior change.

Third, structural interventions are essential. Positive attitudes toward stray animal control expressed by more than 90% of respondents provide a strong mandate for authorities to implement humane catch–neuter–vaccinate–release programs, which have proven effective in Asia and Latin America. Likewise, In Saudi Arabia, rabies PEP is delivered through Ministry of Health services, primarily via public hospitals and primary healthcare centers. Therefore, ‘equitable access’ refers not only to awareness but also to the practical availability of bite management pathways, timely vaccine administration schedules, and access to rabies immunoglobulin when indicated—particularly for rural and remote communities [16].

Regarding governance and programming, Saudi Arabia has ongoing multi-sector efforts relevant to rabies prevention and control (human bite management/PEP pathways, veterinary services, and municipal animal management). Publicly available governmental communications describe a national ‘program to combat rabies’ implemented in coordination with international partners and emphasize cross-sector collaboration consistent with a One Health approach. In addition, national-level workshops convened by relevant authorities have explicitly recommended developing a national plan for rabies control under a One Health framework. However, we did not identify a single publicly accessible document that consolidates these activities into a formally published, stand-alone ‘national rabies elimination programme’ with an explicit operational roadmap and targets. At the regional level, we similarly did not identify a dedicated rabies elimination programme formally led under the Gulf Cooperation Council as a unified initiative; nevertheless, regional coordination mechanisms and technical resources for rabies control and elimination are available through the WOAH Middle East regional platform and the global ‘Zero by 30’ architecture. Future national action would benefit from publishing an explicit One Health rabies strategy/roadmap that defines governance, financing, surveillance, dog/cat vaccination targets, wildlife surveillance, and equitable PEP access—aligned with Zero by 30.

### 4.5. Psychological Dimensions of Rabies Risk

Beyond epidemiology, rabies evokes profound psychological responses due to its association with fear, aggression, and inevitable fatality once symptomatic. Public perceptions of rabies are often shaped by emotional heuristics rather than rational risk appraisal. This may explain why attitudes toward prevention were highly favorable in this study, even though practices lagged behind. Fear-driven attitudes can motivate intention but do not guarantee sustained behavior change unless accompanied by enabling environments and reinforcement.

The reliance on traditional remedies, despite knowledge of rabies severity, reflects cognitive dissonance—individuals attempt to reconcile fear of rabies with culturally familiar practices that provide a sense of control. Addressing such cognitive conflicts requires culturally sensitive interventions that validate community beliefs while simultaneously promoting biomedical practices. Behavioral change communication strategies should therefore combine fear appeals with efficacy-enhancing messages to avoid fatalism or resistance.

A practical One Health roadmap for rabies in Saudi Arabia would include: (1) harmonized human–animal case definitions and routine data sharing between MoH, veterinary authorities, and municipalities; (2) standardized bite-case management protocols and PEP referral pathways across regions; (3) targeted risk communication for youth, families, and animal handlers emphasizing immediate wound washing and urgent PEP after bites; (4) strengthened animal vaccination strategies (owned pets and high-risk animal populations) alongside humane stray animal management; and (5) wildlife surveillance in areas where wild carnivores may sustain transmission cycles. Such coordinated interventions address both community behavior and system-level access barriers.

Operationalizing One Health for rabies in Saudi Arabia requires coordinated governance across human health (standardized bite management and PEP delivery), veterinary services (mass dog/cat vaccination, outbreak investigation, and responsible pet ownership systems), municipalities (humane stray animal management integrated with vaccination and surveillance), and wildlife/environment stakeholders (wild carnivore surveillance and risk communication). In parallel with system-level coordination, public education is a core elimination pillar. A sustainable approach is to integrate rabies prevention into school health education (age-appropriate bite avoidance, first aid, and prompt care-seeking), while using World Rabies Day as an annual high-visibility opportunity for culturally tailored mass awareness campaigns that reinforce consistent messages on wound washing, urgent PEP access, and community participation.

### 4.6. Strengths and Limitations

This study’s strengths include its large, geographically diverse sample and the use of a validated KAP instrument adapted for the Saudi context. The inclusion of more than 2100 participants provided robust statistical power for both descriptive and inferential analyses. Nonetheless, several limitations warrant consideration. The cross-sectional design precludes causal inference, and the use of online surveys may have introduced selection bias, favoring younger, urban, and technologically literate populations. Self-reported practices are subject to recall and social desirability bias, which may have inflated reports of preventive behaviors. Furthermore, perspectives from remote communities without internet access may be underrepresented. Future studies should incorporate stratified random sampling and mixed-method designs, including qualitative interviews, to capture the nuanced cultural beliefs shaping rabies-related behaviors.

Second, the online convenience/snowball sampling strategy may introduce selection bias and network homophily, whereby participants’ referrals are more likely to include individuals with similar sociodemographic characteristics, potentially limiting sample diversity and generalizability. To mitigate this, we disseminated the survey link across multiple platforms (e.g., WhatsApp, X/Twitter, Instagram) and multiple regional channels to broaden reach beyond a single network, and we reported results stratified by key sociodemographic variables with multivariable models to partially account for measured differences. However, individuals with limited digital access, low literacy, or low engagement with social media may remain under-represented; therefore, findings should be interpreted as geographically nationwide in coverage rather than nationally representative.

Finally, mediation models estimated from cross-sectional data should be interpreted as associational (hypothesis-generating) rather than causal pathways

## 5. Conclusions

In summary, this study identifies critical gaps in rabies knowledge and preventive practices among the Saudi population, despite generally positive attitudes toward prevention. Younger individuals, lower-income groups, and residents of the Northern region were particularly vulnerable, reflecting sociodemographic disparities in health behavior. Knowledge was found to mediate the relationship between demographic variables and practices, underscoring the importance of educational interventions. To achieve the global *Zero by 30* target, Saudi Arabia must strengthen rabies literacy, integrate culturally tailored health promotion, ensure equitable access to post-exposure prophylaxis, and implement sustainable animal control programs. A multi-sectoral, One Health approach that addresses both psychological and structural determinants of behavior is essential to close the existing prevention gaps and protect public health.

## Figures and Tables

**Table 1:** Sociodemographic Characteristics of Participants (*n* = 2116).

Variable	Category	Frequency (*n*)	Percentage (%)
Age	Less than 25	1345	63.6
	25–45	642	30.3
	More than 45	129	6.1
Gender	Female	1620	76.6
	Male	496	23.4
Education level	School	327	15.5
	University	1630	77.0
	Postgraduate	159	7.5
Occupation	Student	1223	57.8
	Employee	530	25.0
	Unemployed	363	17.2
Social status	Married	549	25.9
	Unmarried	1567	74.1
Residency	Northern region	338	16.0
	Southern region	276	13.0
	Central region	680	32.1
	Eastern region	362	17.1
	Western region	460	21.7
Family income	Enough and safe	327	15.5
	Enough	1452	68.6
	Not enough	337	15.9
Pet ownership	No	1668	78.8
	Yes	448	21.2
Area has stray animals	No	1044	49.3
	Yes	1072	50.7
Ever bitten by an animal	No	1894	89.5
	Yes	222	10.5
Heard of/known someone with rabies	No	1254	59.3
	Yes	588	37.8
	I don’t know	274	12.9

**Table 2:** Knowledge About Rabies Among Participants (*n* = 2116).

Knowledge Item	Response	*n*	%
Is rabies an infectious disease?	Yes	579	27.4
	No	576	27.0
	I don’t know	961	45.4
Is rabies preventable?	Yes	1351	63.8
	No	83	3.9
	I don’t know	682	32.2
How can rabies be prevented?	Avoid wild animals	529	25.0
	Vaccination	780	36.9
	Good hygiene after animal contact	414	19.6
	I don’t know	393	18.6
What is the cause of rabies?	Viral	882	41.7
	Bacterial	457	21.6
	Bad food or poison	61	2.9
	I don’t know	716	33.8
Which animals can spread rabies?	Dog	1431	67.6
	Cat	12	0.6
	Camel	10	0.5
	Wolf	11	0.5
	All listed animals	374	17.7
	I don’t know	278	13.1
Transmission route of rabies	Bite	1704	80.5
	Scratch	61	2.9
	Inhaling aerosols with viral particles	67	3.2
	I don’t know	284	13.4
Symptoms of rabies in humans	Excessive salivation	377	17.8
	Aggressive behavior	379	17.9
	Fear of water (hydrophobia)	556	26.3
	Barking	109	5.2
	I don’t know	695	32.8
Symptoms of rabid animals	Eye discoloration	121	5.7
	Aggressive behavior	1115	52.7
	Loss of hair	109	5.2
	I don’t know	771	36.4
Incubation period of rabies	Less than 14 days	400	18.9
	1–3 months	227	10.7
	More than a year	79	3.7
	I don’t know	1410	66.6
Outcomes of untreated rabies	Easy to treat	123	5.8
	High mortality	843	39.8
	Always fatal	308	14.6
	I don’t know	842	39.8
What should be done after an animal bite?	Wash wound with soap and water ≥ 15 min	303	14.3
	Use local herbs	42	2.0
	Visit hospital for vaccination	1309	61.9
	Do nothing	21	1.0
	I don’t know	441	20.8
Total knowledge score (Mean ± SD)		28.04 ± 7.57	—
Knowledge classification	Good knowledge	955	45.1
	Poor knowledge	1161	54.9

**Table 3:** Attitudes Toward Rabies Prevention (*n* = 2116).

Statement	Agree *n* (%)	Neutral *n* (%)	Disagree *n* (%)
Rabies is a human health risk	1918 (90.7)	167 (7.9)	31 (1.5)
Controlling stray animals is vital	1908 (90.1)	166 (7.8)	42 (1.9)
Pet vaccination prevents rabies	1774 (83.8)	282 (13.3)	60 (2.9)
Immediate hospital visit after bite	1893 (89.5)	181 (8.6)	42 (2.0)
Vaccination not needed if wound minor	751 (35.5)	415 (19.6)	950 (44.9)

**Table 4:** Frequency Distribution of Rabies-Related Practices Among Study Participants (*n* = 2116).

Practice Variable	Always *n* (%)	Often *n* (%)	Sometimes *n* (%)	Never *n* (%)
Need someone to remind you to get vaccinated when bitten	985 (46.4)	505 (23.8)	307 (14.5)	319 (15.0)
Advise bitten victim to be vaccinated after suspected rabid bite	1444 (68.1)	469 (22.1)	161 (7.6)	42 (2.0)
Play with stray pets	407 (19.2)	215 (10.1)	342 (16.1)	1152 (54.3)
Keep away from aggressive animals	1607 (75.8)	203 (9.6)	116 (5.5)	190 (9.0)
Wash wound with soap and water ≥ 15 min	1146 (54.0)	457 (21.5)	257 (12.1)	256 (12.1)
Use local herbs on the bite	415 (19.9)	346 (16.3)	416 (19.6)	939 (44.3)
Visit the hospital for vaccination	1654 (78.0)	285 (13.4)	139 (6.6)	38 (1.8)
Do nothing and let the wound heal	301 (14.2)	169 (8.0)	182 (8.6)	1464 (69.0)

Total practice score (Mean ± SD): 18.14 ± 7.46; Good practice: 1403 (66.3%); Poor practice: 713 (33.7%).

**Table 5:** Inferential Analysis of Factors Associated with Knowledge, Attitudes, and Practices Regarding Rabies (*n* = 2116).

Predictor Variable	Knowledge (Good vs. Poor)	Attitudes (Positive vs. Negative)	Practices (Good vs. Poor)
Age < 25 years	OR = 0.82 (95% CI: 0.64–1.05), *p* = 0.112	OR = 0.94 (95% CI: 0.71–1.25), *p* = 0.676	OR = 0.61 (95% CI: 0.46–0.82), *p* = 0.001 *
Female gender	OR = 1.07 (95% CI: 0.84–1.35), *p* = 0.596	OR = 1.12 (95% CI: 0.87–1.44), *p* = 0.367	OR = 0.92 (95% CI: 0.73–1.17), *p* = 0.509
University education	OR = 1.28 (95% CI: 0.93–1.76), *p* = 0.121	OR = 1.19 (95% CI: 0.84–1.69), *p* = 0.321	OR = 1.16 (95% CI: 0.84–1.61), *p* = 0.370
Higher income (enough & safe)	OR = 2.14 (95% CI: 1.62–2.84), *p* < 0.001 *	OR = 1.34 (95% CI: 1.02–1.76), *p* = 0.041 *	OR = 1.48 (95% CI: 1.14–1.93), *p* = 0.003 *
Pet ownership	OR = 1.73 (95% CI: 1.29–2.31), *p* < 0.001 *	OR = 1.18 (95% CI: 0.88–1.58), *p* = 0.265	OR = 1.42 (95% CI: 1.07–1.87), *p* = 0.014 *
Central region	OR = 1.56 (95% CI: 1.18–2.06), *p* = 0.002 *	OR = 1.22 (95% CI: 0.91–1.63), *p* = 0.171	OR = 1.41 (95% CI: 1.05–1.88), *p* = 0.020 *
Northern region	OR = 0.71 (95% CI: 0.53–0.96), *p* = 0.025 *	OR = 0.84 (95% CI: 0.61–1.16), *p* = 0.287	OR = 0.58 (95% CI: 0.43–0.77), *p* < 0.001 *

* Significant at *p* < 0.05.

## Data Availability

The data that support the findings of this study are not publicly available due to ethical and privacy restrictions, but are available from the corresponding author on reasonable request.
